# Aseptic loosening around total joint replacement in humans is regulated by miR-1246 and miR-6089 via the Wnt signalling pathway

**DOI:** 10.1186/s13018-024-04578-2

**Published:** 2024-01-29

**Authors:** Yi Deng, Kate Phillips, Zhi-Ping Feng, Paul N. Smith, Rachel W. Li

**Affiliations:** 1 Australian National University Medical School, Canberra, Australia; 2 Department of Orthopaedic Surgery, Canberra Hospital, Canberra, Australia; 3 John Curtin School of Medical Research, Australian National University, Canberra, Australia

**Keywords:** Aseptic loosening, MicroRNA, Sequencing, Wnt pathway, Revision surgery

## Abstract

**Background:**

Total joint replacement for osteoarthritis is one of the most successful surgical procedures in modern medicine. However, aseptic loosening continues to be a leading cause of revision arthroplasty. The diagnosis of aseptic loosening remains a challenge as patients are often asymptomatic until the late stages. MicroRNA (miRNA) has been demonstrated to be a useful diagnostic tool and has been successfully used in the diagnosis of other diseases. We aimed to identify differentially expressed miRNA in the plasma of patients with aseptic loosening.

**Methods:**

Adult patients undergoing revision arthroplasty for aseptic loosening and age- and gender-matched controls were recruited. Samples of bone, tissue and blood were collected, and RNA sequencing was performed in 24 patients with aseptic loosening and 26 controls. Differentially expressed miRNA in plasma was matched to differentially expressed mRNA in periprosthetic bone and tissue. Western blot was used to validate protein expression.

**Results:**

Seven miRNA was differentially expressed in the plasma of patients with osteolysis (logFC >|2|, adj-*P* < 0.05). Three thousand six hundred and eighty mRNA genes in bone and 427 mRNA genes in tissue samples of osteolysis patients were differentially expressed (logFC >|2|, adj-*P* < 0.05). Gene enrichment analysis and pathway analysis revealed two miRNA (miR-1246 and miR-6089) had multiple gene targets in the Wnt signalling pathway in the local bone and tissues which regulate bone metabolism.

**Conclusion:**

These results suggest that aseptic loosening may be regulated by miR-1246 and miR-6089 via the Wnt signalling pathway.

## Introduction

Aseptic loosening around total joint replacements (TJAs) remain an ongoing challenge. Despite the success of TJA, aseptic loosening still accounts for more than 20% of all revision procedures for primary total hip and knee arthroplasty [1]. The pathophysiology of aseptic loosening is due to prosthetic wear particles eliciting an inflammatory response. This causes an imbalance in the activity of osteoclasts and osteoblasts, eventually leading to osteolysis, which may result in component loosening and pain or instability. [2]. However, this theory alone does not explain why some patients have significant wear of the prosthesis but very minimal osteolysis and vice versa. Perhaps altered gene expression in response to wear particles can predispose certain patients to osteolysis and subsequent aseptic loosening [3, 4].

Aseptic loosening can be difficult to diagnose. Early changes are often undetected due to limitations in our current diagnostic modalities. By the time patients are symptomatic, the prosthesis may have already failed, therefore often necessitating a revision procedure [5]. Currently, there is no gold-standard diagnostic test for aseptic loosening. A combination of clinical features, radiographs, cross-sectional imaging and nuclear medicine studies can be used [6]. Biochemical markers have been trialled in the past with varying results [7]. Given the multiple challenges in diagnosis and treatment, it is important to discover novel methods of diagnosis and treatment.

MicroRNA (miRNA) and small interference RNA (siRNA) are a contemporary subject of investigation, which shows promise as biomarkers and therapeutic targets [8–11]. MicroRNA is a family of small, non-coding RNA which regulates gene expression at the post-transcriptional level. They can silence genes via repressing translation or direct cleavage of messenger RNA (mRNA) [12]. There have also been reports of select miRNA upregulating target genes as an alternative method of regulation [13]. Several miRNA has been implemented in the development of diseases of the musculoskeletal system [14, 15]. MiRNA is readily detectable in bodily fluids such as blood and urine inside microvesicles known as exosomes. These exosomes protect miRNA from degradation by RNases which makes them useful as a candidate for biomarkers [16]. Furthermore, understanding the function and gene targets of miRNA can potentially lead to the development of a new class of gene therapy drug [12, 15].

Several miRNA have been shown to regulate osteogenesis and bone metabolism [17]. In vitro studies demonstrate miR-24 has an important role in osteoclast differentiation in the presence of titanium particles [18]. In vivo studies have linked miR-21 and miR-130b to osteolysis due to metal particles in mice [19, 20]. In humans, miR-106b has a role in osteoclastogenesis and osteolysis in bone tumours [21]. However, there are no studies to our knowledge exploring the role of miRNA in human patients with aseptic loosening using next-generation sequencing. A genetic test to predict the likelihood of developing aseptic loosening will allow clinicians to stratify those at risk of the disease and monitor them more closely. It can also be utilised to help diagnose patients who have the disease before any radiographic signs develop. Identifying genes which are responsible for causing increased bone resorption around the implant can be the foundations of future genetic, patient-specific therapies. Thus, we aimed to identify the miRNA expression profile in plasma of patients undergoing revision for aseptic loosening compared to controls. We hypothesise that the miRNA expression profile in patients with aseptic loosening will be different compared to controls, and that these differentially expressed miRNA have mRNA targets in the bone and tissue of aseptic loosening patients.

## Materials and methods

### Study cohort

Adult patients undergoing elective hip or knee arthroplasty across two centres were recruited over an 8-year period. Cases were patients undergoing revision arthroplasty for aseptic loosening (RAL), and controls were patients undergoing primary TJA for osteoarthritis (PA). The diagnosis for aseptic loosening was made by the senior orthopaedic surgeon performing the procedure using clinical findings, radiographs and CT or MRI scans demonstrating loosening or periprosthetic osteolysis. The control group was age and gender-matched patients who were undergoing primary TJA for osteoarthritis. Exclusion criteria included active infection, bloodborne viruses, inflammatory arthritis, immunomodulatory drugs, osteoporosis and metabolic bone diseases. Demographic data were collected including age, sex, BMI, ethnicity, joint affected, comorbidities, immunomodulatory medications, biochemistry studies and the Charlson Comorbidity Index.

Ethics approval was granted by the ACT Health Research Ethics and Governance Office, Human Research Ethics Committee (ETH.9.07.865) to recruit patients and collect intraoperative tissue samples and blood samples. Written informed consent was obtained from each patient.

#### RNA sequencing

### Sample collection and RNA extraction

For RAL patients, intraoperative samples of bone and soft tissue around the prosthesis were collected by the primary surgeon. Bone samples were taken from around the areas of loosening and osteolysis. Soft tissue samples were taken from the capsule and pseudomembrane around the joint. For PA patients, intraoperative samples of bone and soft tissue around the hip or knee joint were collected by the primary surgeon. For the hip, bone was removed from the femoral head and for the knee, bone was removed from the cut surfaces of bone. Soft tissue samples were obtained from the capsule of the hip and knee joint. Samples were collected using a surgical knife, surgical curettes and/or Rongeurs and were stored in sterile containers on ice until processing. Blood samples were collected from an intravenous cannula or arterial line during the procedure and stored in EDTA tubes in ice until processing. All blood samples were processed within 1 h of collection to minimise degradation of RNA.

### Bone and tissue

Bone and tissue samples were cut into small 1mm^3^ pieces under aseptic technique. Care was taken to remove areas of heat necrosis. Specifically for bone, we harvested bone away from the cut surface such as the centre of the femoral head. For tissue, we removed areas damaged by electrocautery and only used samples which were not visibly necrotic. These were snap frozen using liquid nitrogen and homogenised using a mortar and pestle. Total RNA was extracted from each sample using TRIzol reagent and the RNeasy Mini Kit (QIAGEN, USA) according to the manufacturer’s protocol. Samples which were not immediately processed were stored fresh in -80°C in sterile Eppendorf tubes until future use.

### Plasma

Whole blood was centrifuged using two cycles to separate plasma and remove cellular debris. Samples with visible haemolysis were discarded. Two hundred microlitres of plasma were used for miRNA isolation using the miRNeasy Serum/Plasma Kit (QIAGEN, USA) according to the manufacturer’s protocol.

Purified samples of RNA were stored in microfuge tubes and frozen at − 80°C until further use. The quality and concentration of RNA isolated was measured by the NanoDrop Spectrophotometer (ThermoFisher, USA), and the highest quality samples were used for quantification using the Bioanalyzer 2100 (Agilent, USA). Samples with an RNA Integrity Number > 7 were used for sequencing.

### RNA sequencing

A cDNA library was synthesised from RNA samples and sequencing was conducted using a NovaSeq 6000 Sequencing System (Illumina, USA) with a 100bp run. We refer to an individual deep sequence read as a tag and the number of times it occurs as a count. The absolute counts for each miRNA were recorded onto a spreadsheet and those miRNA with very low counts (i.e. < 10) were excluded.

### Statistics

Demographic data were analysed using SPSS 26 (IBM, USA). Chi-square tests were used to compare categorical variables and a student *t*-test for continuous variables. A *P*-value of < 0.05 was deemed statistically significant.

#### Sequencing data analysis

For tissue and bone total RNA samples, data analysis was performed using Bioconductor software in the R programming language via Rstudio. Single-end raw reads were aligned to the human genome, hg38, using hisat2. The mapped reads were assigned based on the NCBI genome annotation with FeatureCounts v2.0. Pre-processing and denoising of the data were performed using edgeR and differentially expressed genes were identified using limma with the voom method. *P*-values were adjusted using the Bonferroni method and an adj-*P* < 0.05 and a logFC >|2.0| was deemed statistically significant. Graphical representations were generated using the Glimma package. Gene-set enrichment analysis was performed with Enrichr and GSEA of C2 and C7 sets in the Molecular Signatures Database and the DisGeNET database. Gene-ontology over-representation testing was performed to identify molecular function, biological processes and cellular components.

For plasma miRNA samples, data analysis was performed using the bioinformatic tools available on Galaxy and the R programming language via RStudio. The quality of the reads was checked with FastQC. Adapters were trimmed using Cutadapt according to the instructions provided in the CATS Small RNA-seq kit (Diagenode, USA). The trimmed reads were mapped to the whole human genome, hg38 using miRDeep2, which utilises Bowtie. Reads with counts less than ten were filtered to minimise false positives. Differentially expressed genes were identified using DESeq2. *P*-values were adjusted using the Bonferroni method and an adj-*P* < 0.05 and a logFC >|2.0| was deemed statistically significant.

To determine miRNA-mRNA target expression pairs, differentially expressed miRNA genes were matched to significantly enriched C3 miRNA target gene sets in the MSigDB. TargetScan Human 7.2 was to predict gene targets of differentially expressed miRNA by matching miRNA targets and C2 canonical pathway gene sets relevant to bone metabolism: inflammation, RANKL, Wnt signalling, JAK-STAT signalling and BMP signalling. Of these, significantly up or down-expressed genes were identified according to a cutoff of adj-*P* < 0.05 and logFC >|2.0|. Furthermore, we applied a stricter criterion using a cutoff of adj-*P* < 0.01 and logFC >|3.0| to identify top target genes relevant to bone metabolism. A manual literature search was performed of the resultant target genes to confirm the genes with direct relevance to bone homeostasis, osteoblast and osteoclast function. These genes were selected for protein expression experiments.

#### Western Blot

### Protein extraction from bone

Intraoperative samples were thawed to room temperature and homogenised using a mortar and pestle with liquid nitrogen. 1 × concentration of RIPA buffer with protease inhibitor at a volume of 10ml RIPA / gram of wet tissue. The samples were centrifuged at 10,000 × g for 15min at −4°C. The supernatant was collected and stored in −20°C until further processing. A Bradford protein assay was performed to determine concentrations of each protein sample.

### Western blot

Protein samples were thawed at room temperature, and 5µg of protein were loaded into each well of the SDS-PAGE gel (12- well TruPAGE Precast Gel 4–12% 8 × 10cm, Sigma-Aldrich, USA). Gels were run for 20min at a constant 50V and at 45min at a constant 80V in Nu-Sep running buffer (Sigma-Aldrich, USA) at room temperature. Proteins were transferred to a PVDF membrane using Nu-Sep Transfer Buffer (Sigma-Aldrich, USA) at 100V for 1 h at room temperature. The PVDF membranes were blocked using blocking buffer and stained using primary and secondary antibodies. The membrane was incubated for 2 h at room temperature with the primary antibody FRAT2 Polyclonal Rabbit anti-Human IgG antibody (Bioss, USA) at a 1:500 dilution. The membrane was incubated for 1 h at room temperature with the secondary antibody Goat anti-Rabbit IgG antibody HRP (abcam, USA) at a 1:2000 dilution. Proteins were stained using the ECL Prime Western Blotting Detection Reagent (Sigma-Aldrich, USA) and detected using an Amersham 680 Imager (GE Life Sciences, USA) via the chemiluminescence method. Images were enhanced using Adobe Lightroom Classic CC (Adobe, USA).

## Results

### Patient demographics

A total of 50 patients were recruited to our study—24 patients in the RAL group and 26 patients in the PA group. There were no significant demographic differences between the patient groups (Table 1). Fifteen patients underwent miRNA sequencing for plasma miRNA and 35 patients underwent total RNA sequencing to identify RNA genes in bone and tissue samples.

**Table 1:** Patient demographics

	Revision *N* = 24	Primary *N* = 26	*p*-value
Age	67 ± 2	72 ± 2	0.31
Sex	6 male 18 female	6 male 20 female	0.81
Joint	22 hip 2 knee	16 hip 10 knee	0.07
Height (m)	1.62 ± 0.03	1.64 ± 0.03	0.78
Weight (kg)	85.2 ± 6.4	74.5 ± 6.1	0.26
BMI (kg/m^2^)	32.3 ± 2.4	27.7 ± 1.7	0.17
Charlson Comorbidity Index	2.9 ± 0.5	2.7 ± 0.3	0.74
Cardiovascular disease	12	14	0.79
Diabetes mellitus	4	4	0.72
Autoimmune disease	0	0	N/A
Cancer	0	0	N/A
Respiratory disease	4	3	0.45
Osteoporosis	0	0	N/A
Renal impairment	2	0	0.18
Immunomodulatory meds	0	0	1.00
Blood tests			
Haemoglobin (g/L)	130 ± 3	134 ± 3	0.36
Creatinine (µmol/L)	74 ± 10	62 ± 5	0.87
eGFR	74 ± 5	80 ± 3	0.94
Calcium (mmol/L)	2.29 ± 0.05	2.29 ± 0.05	0.87
Phosphate (mmol/L)	1.30 ± 0.30	1.36 ± 0.31	0.94
Magnesium (mmol/L)	0.81 ± 0.04	0.72 ± 0.03	0.34
Potassium (mmol/L)	4.69 ± 0.17	4.38 ± 0.10	0.20

### The expression profile of miRNA in plasma of patients with aseptic loosening is significantly different compared to controls

**Fig. 1:**
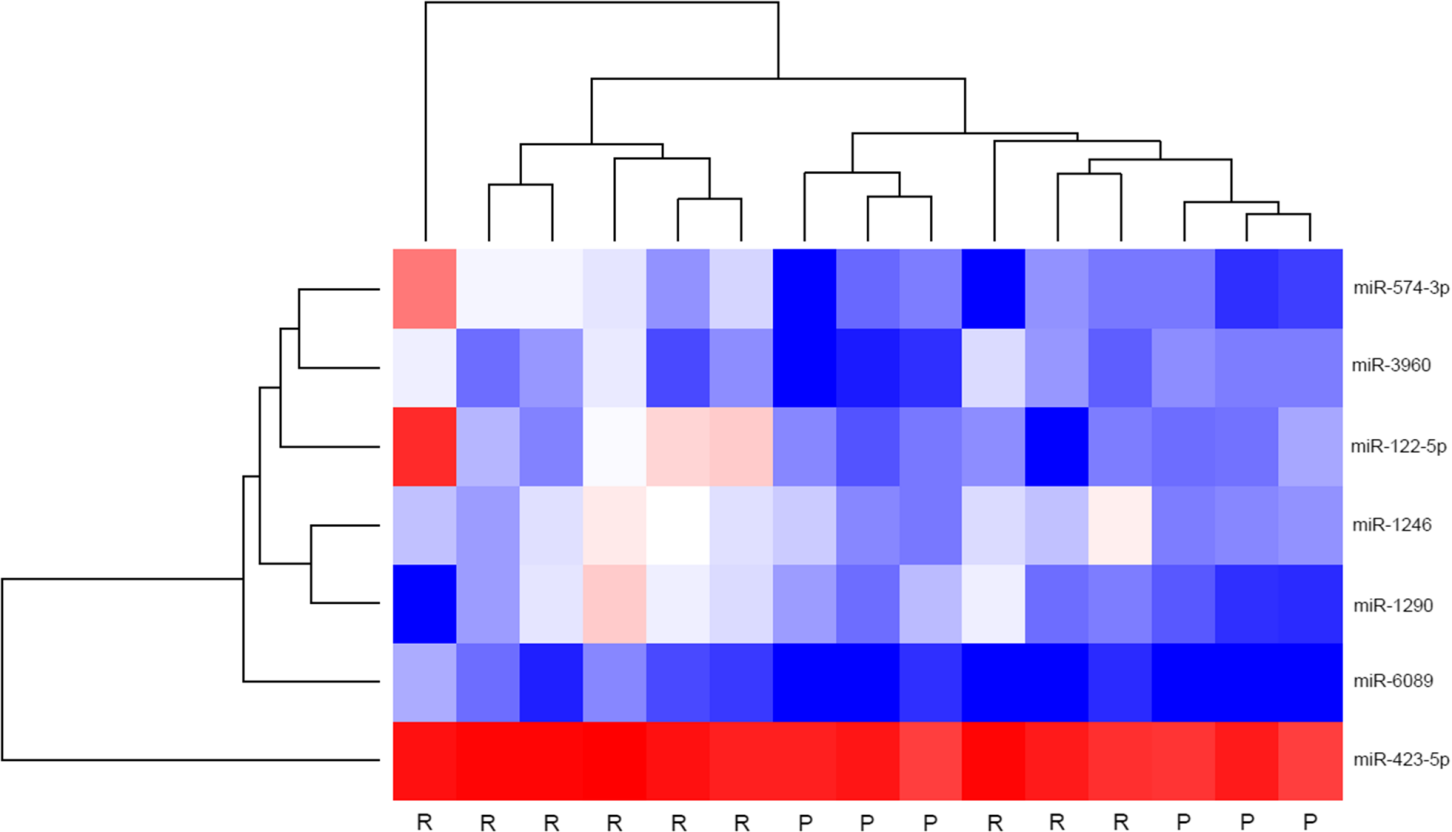
Heatmap demonstrating expression levels of differentially expressed miRNA genes in plasma of aseptic loosening patients compared to controls logFC >|2.0| ranked by adj-*P* value. *R* revision arthroplasty for aseptic loosening; *P* primary total joint arthroplasty (control)

Eighty miRNA genes were identified in the plasma of our patients using miRNA sequencing. Seven miRNA were significantly up-expressed in RAL compared to PA (adj-*P* < 0.05) and six miRNA also had a logFC > 2.0 (Fig. 1).

### The expression profile of mRNA in bone and tissue of patients with aseptic loosening is significantly different compared to controls

**Fig. 2:**
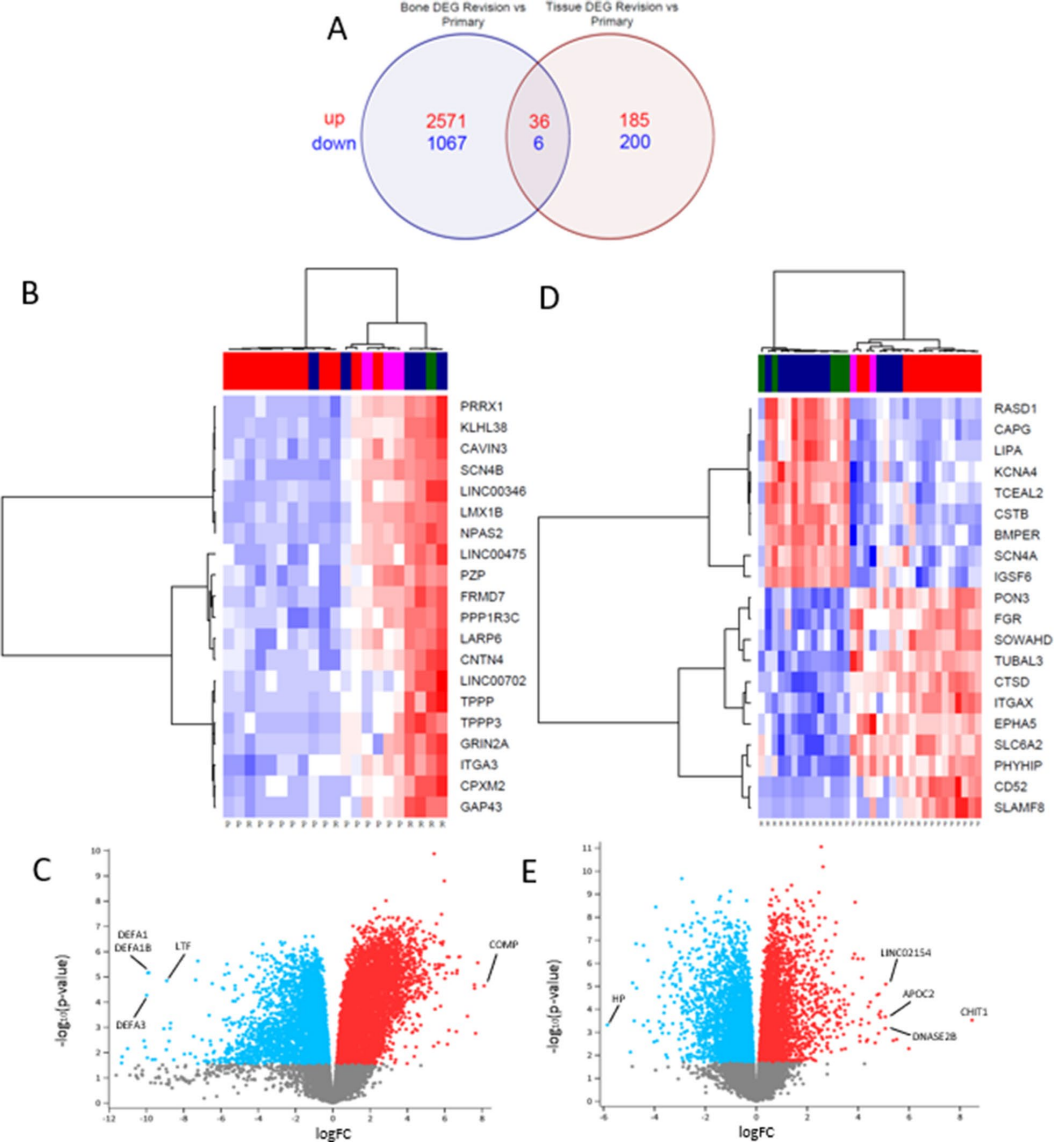
**A** Venn Diagram depicting number of differentially expressed genes in bone and tissue and the number of overlapping genes, up-expressed genes are in red and down-expressed genes in blue; DEG – differentially expressed genes. **B** Heatmap of the top 20 differentially expressed genes in bone samples with logFC >|2.0| ranked by adj-*P* value. **C** Volcano plot of differentially expressed genes in bone samples. **D** Heatmap of top 80 differentially expressed genes in tissue samples with logFC >|2.0| ranked by adj-*P* value. **E** Volcano plot of differentially expressed genes in tissue samples, the top three up or down-expressed mRNA genes are labelled

A total of 3680 differentially expressed genes were identified in the bone of RAL patients compared to PA, of these 2607 were up-expressed and 1173 were down-expressed (adj-*P* < 0.05 and logFC >|2.0|). A total of 427 differentially expressed genes were identified in the tissue of RAL patients compared to controls, of these 221 were up-expressed and 206 were down-expressed (adj-*P* < 0.05 and logFC >|2.0|). Thiry-six genes were up-expressed in both bone and tissue samples and six genes were down-expressed in both bone and tissue samples. The top genes are depicted in Fig. 2 and Table 2.

**Table 2:** Top differentially expressed genes in bone and tissue

Location	Gene	logFC	AveExpr	t	*P* Value	adj.*P*.Val	B
Bone	DEFA1B	− 9.87323	10.52257	− 5.89125	6.82E-06	0.00011	3.695094
	DEFA1	− 9.92204	10.51236	− 5.88224	6.96E-06	0.000111	3.676803
	LTF	− 8.9081	8.370686	− 5.57556	1.42E-05	0.000161	3.187831
	COMP	8.113538	1.880959	5.378271	2.27E-05	0.000213	2.709947
	DEFA3	− 9.98611	8.880224	− 5.01815	5.34E-05	0.000376	1.835942
Tissue	LINC02154	5.085439	−2.17528	5.23428	8.12E-06	0.000152	2.337842
	APOC2	5.065549	0.516944	4.13716	0.000213	0.001447	0.577554
	CHIT1	8.470192	3.545606	4.022007	0.000297	0.001844	0.334267
	HP	− 5.8447	1.932185	-3.85548	0.000479	0.00265	− 0.08945
	DNASE2B	5.064432	− 0.82393	3.731657	0.000681	0.00347	− 0.55021

### In-silico disease gene-set enrichment analysis reveals disease associations with inflammation, osteolysis and osteoporosis

**Fig. 3:**
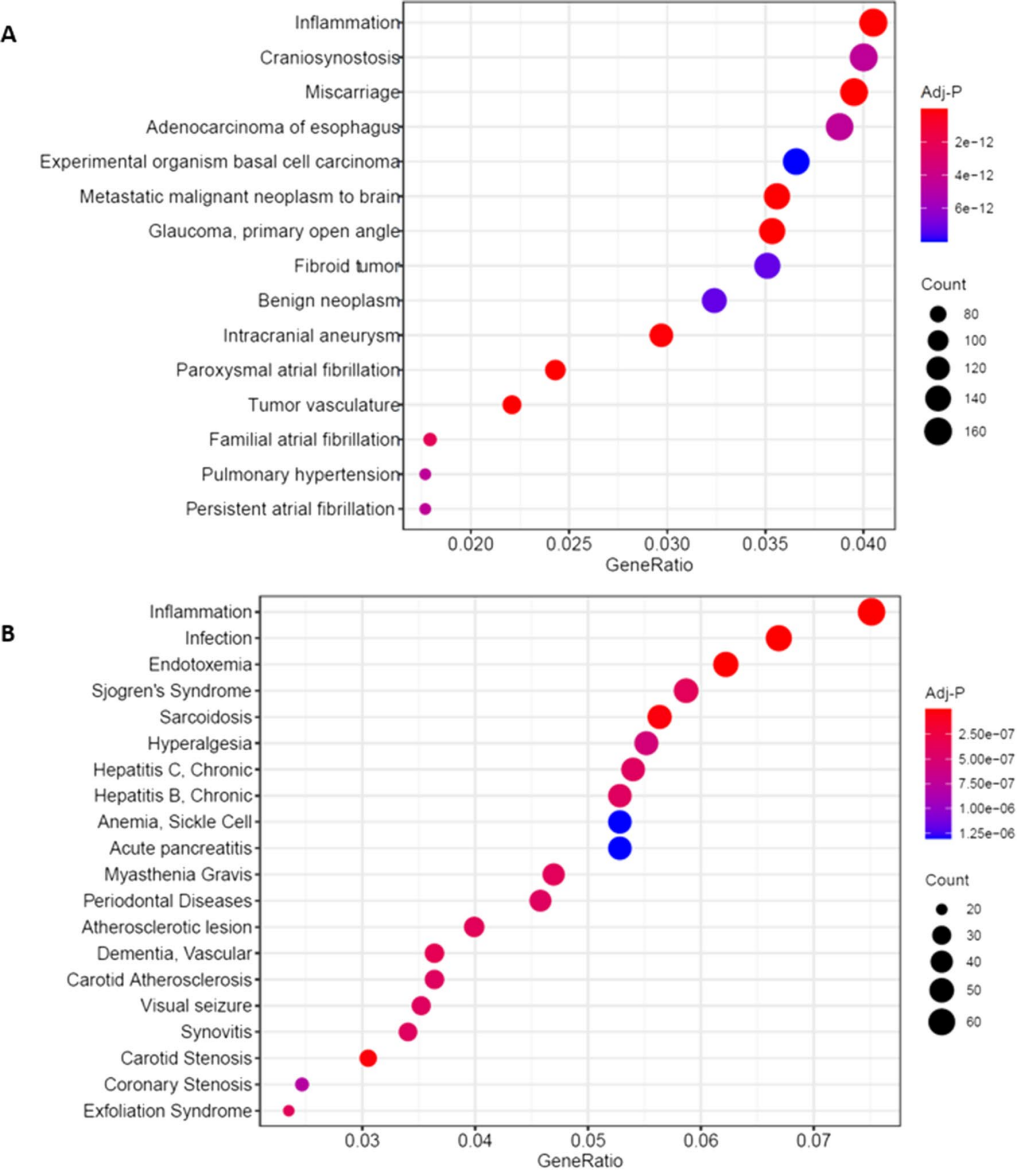
Dot-plot of disease associations using disease gene set enrichment analysis of genes differentially expressed in bone (**A**) and tissue (**B**) samples of patients with aseptic loosening compared to controls

**Fig. 4:**
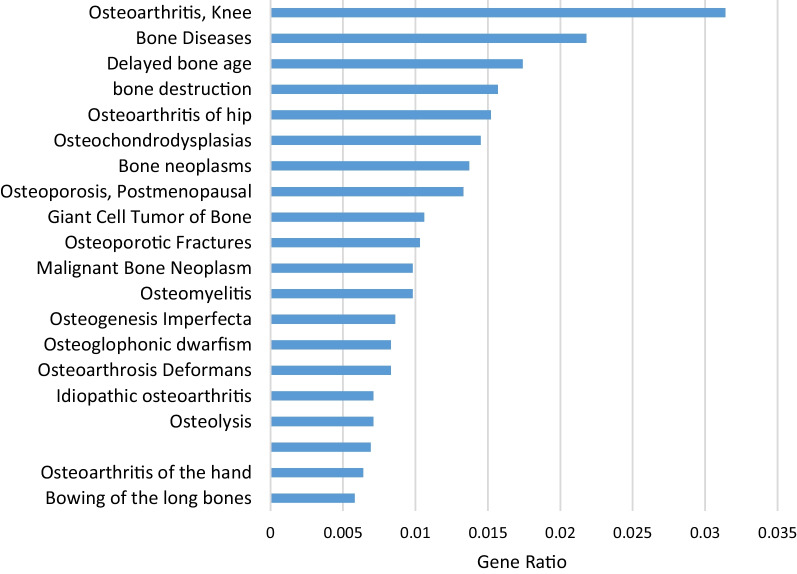
Bone specific disease associations using disease gene set enrichment analysis of differentially expressed genes in patients with aseptic loosening compared to controls

Analysis of gene sets in the DisGeNet 7.0 database demonstrate there is a significant association between patients with osteolysis and the inflammation gene set in both bone and tissue samples (Fig. 3). Of note, synovitis and infection are also in the top 20 disease associations in tissue samples but was not the case for bone samples. Interestingly, RAL patient bone samples were significantly associated with the osteolysis gene set and multiple osteoporosis gene sets. Tissue samples were also associated with multiple osteoporosis gene sets (Fig. 4).

### Target prediction reveals plasma miRNA targets mRNA genes in bone and tissue leading to bone resorption

Analysis of miRNA targets in the C3 regulatory target gene sets in the MSigDB reveals 1116 miRNA in bone samples and 63 in tissue samples which have statistically significant gene sets (adj-*P* < 0.05) in RAL patients compared to controls. Five miRNA identified in our plasma had target gene sets which were significantly enriched in bone samples and two plasma miRNA had target genes which were significantly enriched in tissue samples (Table 3).

**Table 3:** miRNA target prediction using C3 gene sets in MSigDB

	miRNA	GeneRatio	Adj-*P*	Genes
Bone	miR-3960	15/4286	0.00000	PCDHA3/PCDHA10/PCDHA6/PCDHA7/PCDHAC2/PCDHA1 2/PCDHA13/PCDHA2/PCDHA9/PCDHA8/PCDHA11/PCDHAC1/PCDHA5/PCDHA1/PCDHA4
	miR-574-5p	29/4286	0.00014	EPHA3/RGMA/LARP6/RERGL/EVX1/DPP4/IGDCC4/NRN1/C11orf96/TEADl/MDGAl/NEBL/THRB/FOSLl/FOXI2/ADGRB2/MYCBP/SEMA7A/NSUN5/CCDCgsc/RSC1A1/GALNT3/MKRN1/CD9 6/D G KG/ N S D 2/S 0X6/1L5 RA/S10 0 A12
	miR-423-5p	40/4286	0.00091	RIMS4/CADM3/CSMD2/COL1A1/CPEB1/LHX6/ARHGEF4/ TSPAN11/RGMA/KCNIP3/DNAL11/S100A16/NNAT/EVC/C1QTNF6/SYP/ROR2/NAV1/NRSN2/LZTS3/SLC52A3/SLC20A2/CLDN23/ADGRL1/TMEM150A/OBSL1/KLK4/DUSP8/L0C730098/0DC1/CRIP3/C20Orf27/NECTIN1/ATG4D/UBE20/SLC6A9/RAC2/PHOSPHO1/TCLIB/DMTN
	miR-1290	65/4286	0.00166	OGN/KCNK2/DPT/TMEM255A/ANTXR1/COLEC12/ANK2/EFNA5/TGFB 2/COL8A1/SYNP02/THS D7A/RGS5/EGFR/SGIP1/SORCS1/PCDH7/TEAD3/ACKR4/EMP2/PRRG1/RASAL2/CSTB/NAALADL2/VEGFD/GALNT15/PREX2/SRGAP1/ASXL3/ZFHX3/EBF1/ARHGEF26/SEMA6A/AGAP1/ERBB4/KCNAB1/MSR1/LRRTM2/CALCR/DYNC1|1/RASGRF2/TAFA5/CAPN2/HYDIN/MGME1/CAMK4/CBFA2T3/ADARB2/USP15/SMC4/FBN2/SLAIN1/AMMECR1/ITGA4/PIP5K1B/BEND 4/EPCAM/IL5RA/MN D1/PLCH1/SG 01 /TCN1/ MS4A3/SPT A1/TSP02
	miR-122-5p	28/4286	0.00188	STMN2/MYOCD/CPEB1/LALMC1/RANBP3L/MECOM/RBMS2/MAP3K12/DDR2/FLNB/CTNNA3/CLIC5/MIPOL1/KCNN3/MICU3/SLC25A34/CLIC4/ADGRB2/CO40LG/TBC1D22B/S E MA4 D/G6PD/S LC7A1/RBL1/S LC05A1/ PRR11/C21orf62/KIF11
Tissue	miR-3960	7/881	0.00046	PCDHA11/PCDHA2/PCOHA12/PCDHA1/PCDHA10/PCDHAC2/pou3p3
	miR-1290	20/881	0.02309	TMEM255A/CSTB/KLHL6/MS4A3/PLCH1/ARHGEF26/ASXL3/BTG2/THRSP/SORCS1/VEGFD/WNK2/SYN2/GREM2/BEGAIN/CNTN6/ERBB4/SYT4/TRHDE/NRXN1

Target prediction using TargetScan 7.2 identified 432 genes targeted by plasma miRNA in bone samples, of these 88 were differentially expressed in RAL patients (adj-*P* < 0.05, logFC >|2|). In tissue samples, fifty-five genes were targets of plasma miRNA and seven were differentially expressed (adj-*P* < 0.05, logFC >|2|). The top 20 based on adj-*P* are shown in Table 4.

**Table 4:** Top 20 miRNA targets based on adj-*P* value and logFC

miRNA	Bone/tissue	Gene	Direction	Adj-*P*	logFC
miR-1246	Tissue	C7	Down	0.0003	−3.6
	Bone	LINC00346	Up	0.0008	3.3
	Bone	PPP1R3C	Up	0.0008	3.6
	Bone	HOXC10	Up	0.0008	4.0
	Bone	KIAA0930	Up	0.0008	3.6
	Bone	PCDHB2	Up	0.0009	3.3
	Bone	THBS2	Up	0.0009	3.6
	Bone	SIX4	Up	0.0009	3.5
	Bone	LINC00346	Up	0.0008	3.3
	Bone	DIXDC1	Up	0.0008	4.0
	Bone	KLHL30	Up	0.0008	3.2
	Bone	ANTXR1	Up	0.0008	3.3
	Bone	APLN	Up	0.0008	3.3
	Bone	KIAA0930	Up	0.0008	3.6
	Bone	CLDN1	Up	0.0009	3.1
	Bone	PCDHB2	Up	0.0009	3.3
miR-3960_8072	Bone	NTN1	Up	0.0008	3.7
miR-423-5p	Bone	NTN1	Up	0.0008	3.7
miR-574-3p	Tissue	MMP3	Down	0.0005	-3.5
miR-6089	Bone	PPP1R3C	Up	0.0008	3.6
	Bone	ACE	Up	0.0008	3.2
	Bone	DIXDC1	Up	0.0008	4.0
	Bone	KLHL30	Up	0.0008	3.2
	Bone	PPIC	Up	0.0008	3.1
	Bone	NTN1	Up	0.0008	3.7
	Bone	APLN	Up	0.0008	3.3
	Bone	KIAA0930	Up	0.0008	3.6
	Bone	CCDC3	Up	0.0008	3.9

**Fig. 5:**
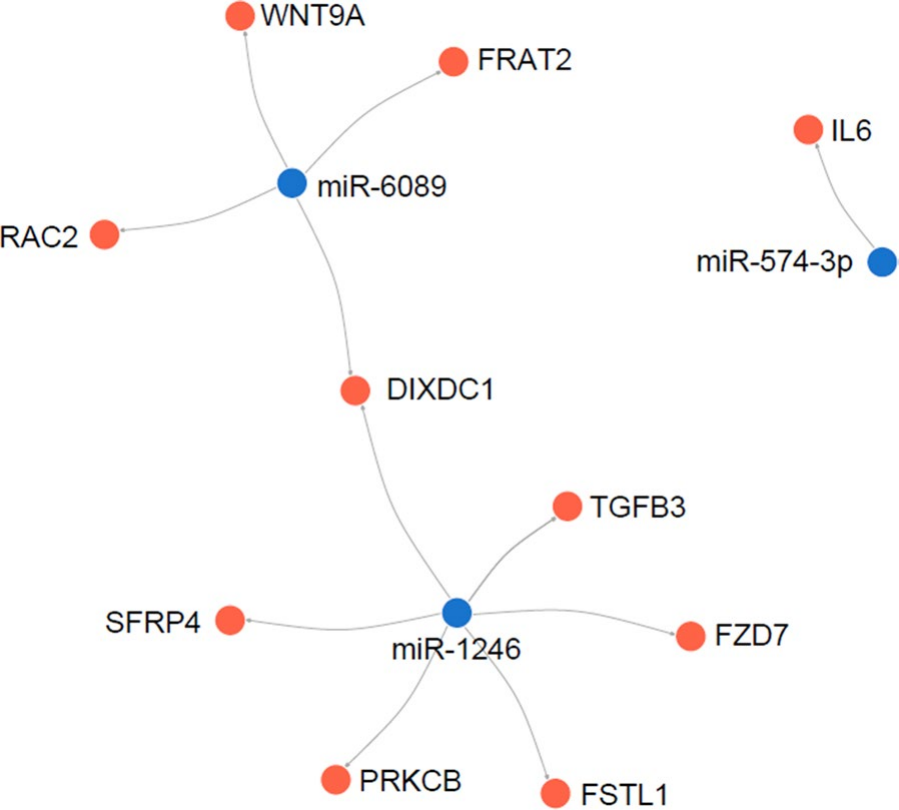
Network visualisation plot demonstrating differentially expressed miRNA in plasma with corresponding targets of differentially expressed genes in the bone of aseptic loosening patients compared to controls

Several gene targets relevant to the C2 pathways relating to inflammation, RANKL, Wnt signalling, JAK-STAT signalling and TGFB signalling were also identified. Seventy-eight genes in bone and 88 genes in tissue were identified which were direct targets of plasma miRNA. Of these, 10 genes in bone and zero genes in tissue were differentially expressed in RAL patients (adj-*P* < 0.05, logFC >|2|) (Table 5 and Fig. 5).

**Table 5:** Differentially expressed miRNA and their gene targets based on C2 pathways

miRNA	Target tissue	Pathway	Gene	Direction	Adj-*P*	logFC
miR-1246	Bone	Wnt	FZD7	Up	0.0038	2.1
	Bone	Wnt	PRKCB	Down	0.0032	-2.5
	Bone	Wnt	DIXDC1	Up	0.0007	4.0
	Bone	Wnt	SFRP4	Up	0.0014	3.6
	Bone	Inflammation	TGFB3	Up	0.0014	2.4
	Bone	TGFB	TGFB3	Up	0.0014	2.4
	Bone	Wnt	FSTL1	Up	0.0037	2.4
miR-574-3p	Bone	JAK-STAT	IL6	Up	0.0080	2.4
miR-6089	Bone	Wnt	RAC2	Down	0.0089	− 2.3
	Bone	Wnt	FRAT2	Down	0.0059	− 2.2
	Bone	Wnt	DIXDC1	Up	0.0008	4.0
	Bone	Wnt	WNT9A	Up	0.0021	2.4

### FRAT2 is down-expressed in patients with aseptic loosening

**Fig. 6:**
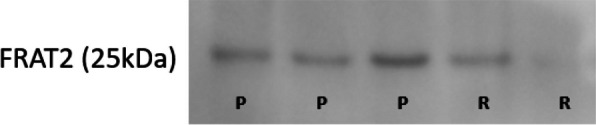
Western blot analysis of FRAT2 protein in bone samples. R revision arthroplasty for aseptic loosening; P primary total joint arthroplasty (control)

Western blot analysis demonstrates FRAT2 is significantly down-expressed in the bone of patients in the RAL group compared to PA group (Fig. 6).

## Discussion

Our results demonstrate that RAL patients have a significantly different gene expression profile in the periprosthetic bone and soft tissue. The expression of miRNA in the plasma of patients with RAL is also significantly different compared to normal controls. Furthermore, these miRNA have targets genes in bone and tissue which are linked to inflammation, bone homeostasis and osteoblast and osteoclast function.

### Upregulation of genes by miRNA could explain aseptic loosening

Interestingly, several miRNA gene targets were upregulated in RAL compared to PA patients (Table 4 and 5). The most widely accepted mechanism of action for miRNA is the silencing of genes by translational repression or by direct cleavage of mRNA [22]. However, there is emerging evidence to suggest other mechanisms of action of miRNA, including the upregulation of genes [23]. In proliferating cells miRNA tend to repress translation whereas in the G_1_/G_0_ arrest phase, they activate translation [24]. There are numerous ways by which miRNA can enhance gene expression, including directly by interactions of miRNA polyribosomes with target genes and indirectly by disengaging inhibitory miRNA from their targets, thereby increasing gene expression [13].

### *Aseptic loosening is regulated by miR-1246 and miR-6089 *via* the Wnt signalling pathway*

Merging gene targets in TargetScan to C2 gene sets in MSigDB reveal that the Wnt signalling pathway had multiple genes that were differentially expressed in RAL patients. Two miRNA, namely miR-1246 and miR-6089 have gene targets in the Wnt pathway (Table 4). Specifically, the Wnt genes targeted by miR-1246 include DIXDC1, SFRP4, PRKCB, FSTL1 and FZD7. The Wnt genes targeted by miR-6089 include DIXDC1, WNT9A, RAC2 and FRAT2.

The Wnt signalling pathway is one of the main pathways regulating bone formation and resorption. The primary role of the canonical Wnt pathway is to stimulate bone formation by activation of osteoblastogenesis [25]. The non-canonical pathway activates osteoclastogenesis and also has implications in inflammatory diseases [26]. Wnt proteins bind to Frizzled (FZD) receptors activating either a β-catenin-dependent canonical pathway or a β-catenin-independent non-canonical pathway, which in-turn regulate downstream genes [27].

Secreted frizzled-receptor proteins (sFRPs) are antagonists of the Wnt pathway by binding directly with FZD receptors, thereby inhibiting the interaction between Wnt and FZD [28]. SFRP4 overexpression has been demonstrated to inhibit osteoblasts, resulting in a 30% decrease in trabecular bone mass in mouse models [29]. Although sFRP4 is implicated in Pyle’s disease, a rare genetic condition characterised by thinning of cortical bone and increased fractures, sFRP4 knockout results in significantly greater trabecular bone mass in mouse models [30]. In patients with osteolysis, there is also a loss of trabecular bone adjacent to the prosthesis, ultimately leading to loosening of the prosthesis [31]. Cortical bone is usually preserved until the later stages. This loss of trabecular bone and preservation of cortical bone is apparent on radiographs as endosteal scalloping [32].

Follistatin-related protein 1 (FSTL1) is encoded by the FSTL gene with a variety of functions in the development of human diseases [33]. It has a pro-inflammatory effect and has been shown to be elevated in chronic inflammatory diseases such as rheumatoid arthritis [34]. Furthermore, overexpression of FSTL1 in bone-marrow derived macrophages positively regulates osteoclast differentiation via RANKL and FSTL1 knockout results in a decrease in osteoclast precursor proliferation [35]. In our RAL patient group, FSTL1 was significantly up-expressed, which suggest FSTL1 has a role in the development of osteolysis and aseptic loosening via an inflammatory response.

FZD7 has been extensively studies in carcinogenesis and has been shown to activate both the canonical and non-canonical Wnt signalling pathways [36]. Non-canonical pathways can lead to osteoclast activation thereby increasing bone resorption [37]. FZD7 was significantly upregulated in AL patients, suggesting a potential role for its role in the pathogenesis of osteolysis and loosening.

FRAT proteins and protein kinase C (PRKCB) are important regulators of the Wnt signal transduction pathway as they bind and deactivate glycogen synthase kinase-3β (GSK-3β) [38, 39]. In the absence of Wnt, GSK-3β phosphorylates β-catenin and stops the activation of the canonical Wnt pathway. Binding of Wnt to FZD receptors results in the phosphorylation and deactivation of GSK-3β, therefore increasing β-catenin, leading to downstream gene regulation [40]. In our AL group, FRAT2 was significantly down-expressed compared to controls, suggesting that FRAT2 downregulation could be implicated in inactivation of the canonical Wnt signalling pathway, leading to decreased osteoblastogenesis.

The RAC proteins are required for normal bone development and homeostasis. Osteoblastic differentiation from mesenchymal stem cells and knockout mice demonstrated marked reductions in bone size and trabecular bone formation [41]. Other groups have demonstrated that RAC deletions lead to increased bone formation due to defects in osteoclastic function [42, 43].

Two genes in the Wnt signalling pathway which did not support our findings were DIXDC1 and Wnt9A. DIXDC1 activates the canonical Wnt pathway as demonstrated in various cancer cell lines [44, 45]. Wnt9A was significantly up-expressed in the periprosthetic bone of RAL patients compared to controls. Wnt9A is crucial for the regulation of endochondral ossification in utero [46]. Wnt9A knockout mice have various bone abnormalities including reduction in bone length and decreased area of mineralised regions. These changes were more pronounced in the proximal bones including the femur and ilium [47]. However, there are no studies to our knowledge which have examined the role of Wnt9A in mature osteoblasts and osteoclasts.

**Fig. 7:**
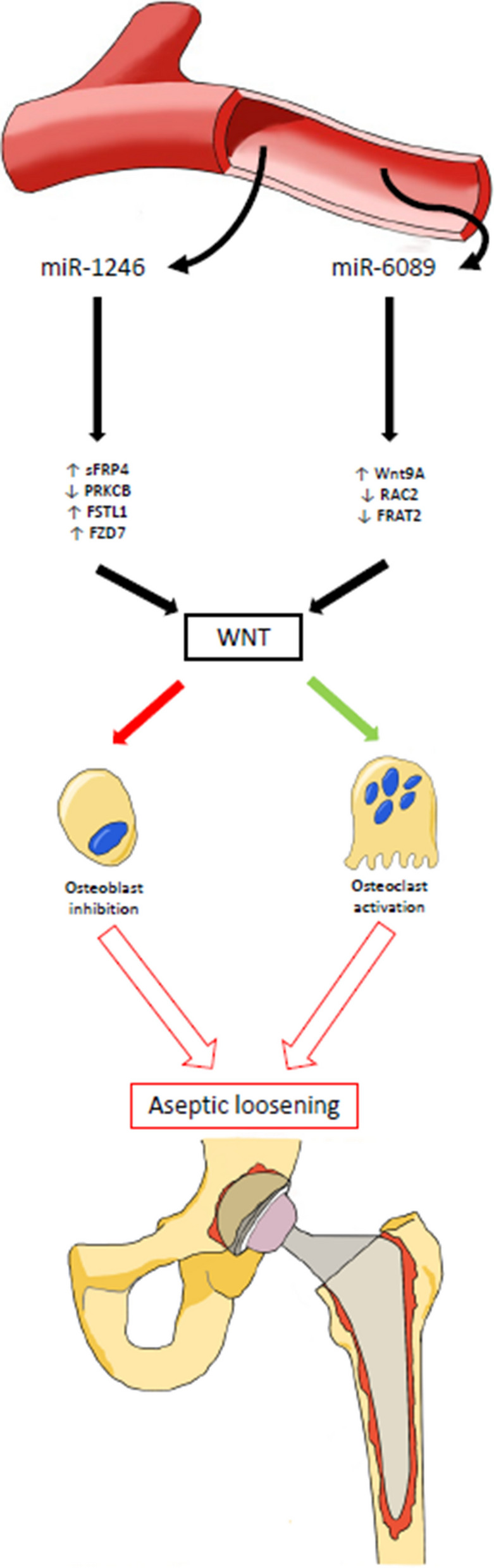
Schematic diagram demonstrating circulating miRNA targeting genes in the periprosthetic bone leading to osteoblast inhibition and osteoclast activation and subsequent aseptic loosening. Circulating miRNA in plasma (miR-1246 and miR-6089) of aseptic loosening patients with target genes in the local periprosthetic bone and soft tissues. These genes, some upregulated, some downregulated, act on the Wnt signalling pathway, which in-turn results in the inhibition of osteoblasts and activation of osteoclasts. Over time, this process results in bone resorption around the prosthesis, osteolysis and aseptic loosening

Taken together, these results suggest that RAL is potentially regulated by miR-1246 and miR-6089 via the Wnt pathway. The genes SFRP4, PRKCB, FSTL1, RAC2, FRAT2 and WNT9A have been demonstrated to affect this process based on previous studies (Fig. 7).

There are several limitations which must be discussed. Firstly, the control group we used to collect bone samples were patients with osteoarthritis undergoing primary TJR. Ideally, the control group would be patients with well-functioning joint replacements not requiring revision surgery or normal healthy patients free of arthritic disease. However, the significant risks associated with taking samples of bone and tissue around a normal joint replacement or from a normal patient preclude us from using this cohort as a control. Secondly, our study cohort had an uneven number of patients having hip arthroplasty and knee arthroplasty. Although there are subtle differences in the disease process of hip and knee arthritis, the underlying biological processes of osteoarthritis and the development of aseptic loosening should remain the same. As hip and knee arthroplasty are the most common forms of arthroplasty performed, we believe combining the two groups make the results more applicable to a wider population. Finally, despite our best efforts, it can be difficult to completely separate bone and tissue samples, especially in revision cases where anatomical layers are distorted, and sample volume is small. This may result in minute amounts of tissue within our bone samples and vice versa.

In conclusion, RAL patients have a significantly different transcriptome compared to controls. Furthermore, there are several miRNA which are detectable in the blood of RAL patients which have gene targets in the bone and soft tissue around the prosthesis. Our findings allow us to understand the role of miRNA in the regulation of genes associated with aseptic loosening.

## Data Availability

Datasets and code used for RNA-sequencing data analysis available from the corresponding author by request.
